# Differences in milk metabolites in Malnad Gidda (*Bos indicus*) cows reared under pasture-based feeding system

**DOI:** 10.1038/s41598-021-82412-z

**Published:** 2021-02-02

**Authors:** M. Ashokan, Kerekoppa P. Ramesha, Sweta Hallur, Gayathree Karthikkeyan, Ekta Rana, N. Azharuddin, S. Reshma Raj, S. Jeyakumar, A. Kumaresan, Mukund A. Kataktalware, D. N. Das, T. S. Keshava Prasad

**Affiliations:** 1 Southern Regional Station, ICAR-National Dairy Research Institute, Adugodi, Bangalore, 560030 India; 2 Center for Systems Biology and Molecular Medicine, Yenepoya Research Centre, Yenepoya (Deemed to be University), Mangalore, 575018 India

**Keywords:** Lipids, Physiology

## Abstract

The milk and milk products from cows reared under grazing system are believed to be healthier and hence have high demand compared to milk from cows reared in the non-grazing system. However, the effect of grazing on milk metabolites, specifically lipids has not been fully understood. In this study, we used acetonitrile precipitation and methanol:chloroform methods for extracting the milk metabolites followed by liquid chromatography–tandem mass spectrometry (LC–MS/MS) run to identify the different metabolites between the milk of grazing and non-grazing early lactating Malnad Gidda cows. Various carbohydrates, amino acids, nucleosides and vitamin derivatives were found to be differentially abundant in grazing cows. A total of 35 metabolites were differentially regulated (fold change above 1.5) between the two groups. Tyrosyl-threonine, histidinyl-cysteine, 1-methyladenine, l-cysteine and selenocysteine showed fold change above 3 in grazing cows. The lipid profile of milk showed a lesser difference between grazing and non-grazing cows as compared to polar metabolites. To the best of our knowledge, this is the largest inventory of milk metabolomics data of an Indian cattle (*Bos indicus*) breed. We believe that our study would help to emerge a field of Nutri-metabolomics and veterinary omics research.

## Introduction

India is blessed with a large number of cattle (193.46 million) population^1^ and ranks first in the world in total milk production (187.7 MT)^2^. Along with quantity, the quality of bovine milk is equally important because it is an important entity in human nutrition as well^3^. The understanding of the molecular composition of bovine milk in different stages and conditions would help in integrative molecular biology and nutrition-based studies^4–7^. Bovine milk composition and quality are mainly influenced by various factors including genetics, diet (feeding system), stage of lactation, season and somatic cell count^4,8^. The difference in the feeding systems significantly influences the milk composition in cows^9–11^. It has been reported that forages like alfalfa hay, corn stover^12^, corn silage, fresh forage and hay^13^ significantly alter the metabolomics profile of the cow milk. Pasture-based (grazing) feeding system is traditionally the most adopted management practice by small-holder dairy farmers (about 86% of all farmers) in India, where the indigenous cattle graze on a natural pasture of native flora. Recently, intensification in the dairy industry resulted in the inclination of the feeding practices towards non-grazing (zero-grazing/stall-fed) system where cows are housed indoor and mainly fed with paddy straw and cultivated green fodder along with concentrates which enables the farmers to have better control over nutrition, health and management of lactating animals^6^.

It has been reported that the nutritional value of paddy straw is lower than alfalfa hay and is a highly lignified material^14^. There is a perception that milk and milk products derived from cows reared in the grazing system are natural and healthier, and hence have high demand in the dairy market^15^. Milk derived from the pasture system has been found to have higher unsaturated fatty acids and more levels of α-tocopherol and β-carotene compared to Total Mixed Ration (TMR) diets^16^. O’Callaghan et al.^6^ have also observed the beneficial influence of the grazing system on the nutritional composition of milk and milk products (butter and cheese) both at macronutrient and fatty acid levels (conjugated linoleic acid and omega 3 fatty acid). Therefore, it could be interesting to observe that grazing has a significant influence on the milk metabolomic profiles, some of which might have health benefits. Besides, metabolomics signatures associated with milk of grazing cows could be used to identify potential candidate biomarkers for differentiating the origin of milk-based on the feeding systems. O’Callaghan et al.^6^ have reported that hippuric acid could be a potential biomarker for pasture-derived ruminant milk. However, the literature on alterations in the milk metabolites due to different feeding systems and their association with physiological and metabolic pathways is limited. Recently, the mass spectrometry-based metabolomics approach gained popularity because of its high resolution, sensitivity and specificity in identifying differentially expressed metabolites under different conditions^17^. Although, several researchers have carried out detailed metabolomics investigation of European cattle (*Bos taurus*) milk, nevertheless, the milk metabolomics of Indian cattle (*Bos indicus*) breeds are yet to be explored. Malnad Gidda (*Bos indicus*), a dwarf cattle breed, mostly reared under a pasture-based free-range system in the Western Ghats and coastal ecosystem of Karnataka, India; which are endowed with rich floral biodiversity including grasses, shrubs and herbs. Interestingly, the milk of Malnad Gidda cattle under grazing system of feeding is highly preferred among the consumers and local traditional healers, with the perception that milk would contain better nutraceutical property^18^. Therefore, the present study was conducted on Malnad Gidda cows to see the effect of pasture (grazing) and stall-fed (non-grazing) based feeding management systems on milk metabolites.

## Results

### Milk metabolomics profiling

**Figure 1:**
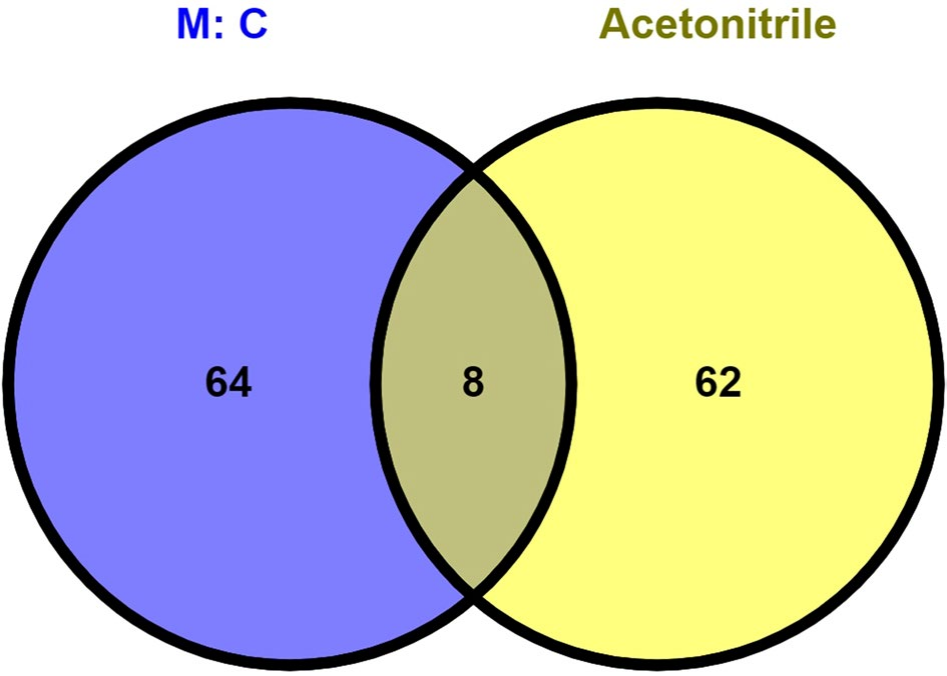
Unique and mutual metabolites selected in methanol:chloroform and acetonitrile method. M:C (methanol:chloroform).

Metabolite profiling of Malnad Gidda milk was performed using liquid chromatography-based tandem mass spectrometry (LC–MS/MS) analysis. The metabolites were searched at both precursor and fragment-level using the m/z list from both XCMS and MZmine 2.5 data processing protocols, which helped in the identification and assignment of a large number of metabolites, using the HMDB and LipidMaps databases. XCMS analysis revealed the identification of 7586 aligned peaks from the acetonitrile method and 14,461 aligned peaks using the methanol-chloroform method (1345 ions in the upper phase and 13,116 ions in the lower phase) using the XCMS tool. Collectively, 134 metabolites were identified between acetonitrile precipitation and methanol:chloroform methods. The species of identified metabolites differed between acetonitrile precipitation and methanol:chloroform methods. Valine, methionine, thiamine monophosphate, ribose 1,5 biphosphate, selenocysteine, selenomethionine and other 2 were detected in both the methods. Thyronine, leucine, cysteine and other 62 metabolites were detected only in the acetonitrile precipitation method, while, glycyl methionine and other 64 metabolites were detected only in the methanol:chloroform method (Fig. 1). The metabolite profiles of milk have been given in the supplementary Table S1 and S2 and animal details given in supplementary Table S3.

Similarly, peak alignment using MZmine for the features with MS/MS-level information resulted in the identification of 11,256 and 18,752 aligned peaks from the positive and negative modes, respectively of the acetonitrile extraction method. The metabolite extraction with the methanol-chloroform method yielded 14,332 and 4973 aligned peaks, from the lower and upper phases of the extraction method. The metabolites assigned at the fragment-level were considered for further analysis. The metabolite assignment at MS/MS-level showed the assignment of 884 (positive mode), and 2967 (negative mode) metabolites from the acetonitrile method, and 174 and 1784 metabolites from the upper and lower phases of the methanol-chloroform method. A total of 2327 non-redundant metabolites were identified, of which 223 were found common to both the modes. A total of 1162 and 942 metabolites were uniquely identified in the methanol-chloroform and acetonitrile methods, respectively. The complete list of metabolite assignment to the respective m/z, retention time, and their peak areas, are given in Supplementary Tables S4–S7. Despite metabolite mapping at different levels, a substantial amount of the m/z features were not assigned to a known metabolite, therefore we are reporting the list of unassigned m/z features, which will pave the way for their identification in the future (Supplementary Tables S8–S11). We primarily focused on the alterations in the amino acids and derivatives, carbohydrates, vitamin derivatives and nucleosides across the grazing and non-grazing cow’s milk.

The quality of the data acquisition was assessed by comparing the sample runs with the intermediate blank runs^19^. The principal component analysis (PCA) showed a clear clustering of the blank runs and group-wise clustering of the sample runs. The PCA analysis also showed that there was no carryover of the samples. Multivariate analysis of the blank and sample runs showed that the important features identified using the variable importance in projection (VIP) scores from the partial least squares differential analysis (PLS-DA) were not identified in the blank runs (Supplementary Fig. S1).

### Milk lipidomics profiling

Lipids extraction was performed by the methanol: chloroform method. A total of 39 lipids from six different classes had been identified in Malnad Gidda milk viz*.* 7 ceramides (Cer), 2 sphingomyelin (SM), 9 phosphatidylcholines (PC), 12 phosphatidylserine (PS), 2 phosphatidylinositol (PI) and 7 phosphatidylethanolamine (PE). Sphingomyelins and ceramides were classified under sphingolipids class. Similarily, phosphatidylcholine, phosphatidylserine, phosphatidylinositol and phosphatidylethanalomine were classified under glycosphingolipids. Among these six classes of lipids, phosphatidylcholine and phosphatidylserine were the most abundant classes of lipids, where as, phosphatidylinositol and sphingomyelin were the least abundant classes of lipids. The identified various lipid classes along with their input mass (m/z ratio), matched mass (m/z ratio), delta error and formula are presented in Table 1.

**Table 1:** Detail of lipid class obtained in the Malnad Gidda cow’s milk.

Lipids	Input mass	Matched mass	Delta	Formula	Ion	Rt (min)
Cer(t40:2)	636.6	636.59	0.008	C_40_H_77_NO_4_	[M + H]+	3.82
Cer (d41:0)	638.64	638.64	0.005	C_41_H_83_NO_3_	[M + H]+	4.17
Cer(m42:2)	632.64	632.63	0.006	C_42_H_81_NO_2_	[M + H]+	4.31
Cer(d42:1)	650.64	650.64	0.005	C_42_H_83_NO_3_	[M + H]+	3.77
Cer(t38:2)	608.52	608.56	0.041	C_38_H_73_NO_4_	[M + H]+	4.26
Cer(d40:3)	618.6	618.58	0.018	C_40_H_75_NO_3_	[M + H]+	4.09
Cer (t38:0)	612.6	612.59	0.008	C_38_H_77_NO_4_	[M + H]+	3.78
PC (36:2)	786.6	786.60	0.001	C_44_H_84_NO_8_P	[M + H]+	4.75
PC(32:2)	746.52	746.53	0.013	C_40_H_76_NO_9_P	[M + H]+	4.47
PC(30:1)	704.52	704.52	0.003	C_38_H_74_NO_8_P	[M + H]+	3.78
PC(36:7)	776.52	776.52	0.003	C_44_H_74_NO_8_P	[M + H]+	4.90
PC(38:4)	810.6	810.60	0.001	C_46_H_84_NO_8_P	[M + H]+	4.79
PC(28:0)	680.52	680.52	0.003	C_36_H_74_NO_8_P	[M + H]+	5.24
PC(28:1)	676.5	676.49	0.009	C_36_H_70_NO_8_P	[M + H]+	3.77
PC(32:3)	728.52	728.52	0.003	C_40_H_74_NO_8_P	[M + H]+	4.80
PC(34:5)	752.52	752.52	0.003	C_42_H_74_NO_8_P	[M + H]+	5.01
PI(32:5)	817.44	817.45	0.010	C_41_H_69_O_14_P	[M + H]+	4.94
PI(36:5)	857.52	857.51	0.003	C_45_H_77_O_13_P	[M + H]+	3.82
SM(d38:3)	755.64	755.60	0.033	C_43_H_83_N_2_O_6_P	[M + H]+	4.93
SM(d42:3)	811.68	811.66	0.011	C_47_H_91_N_2_O_6_P	[M + H]+	4.60
PS(35:4)	786.48	786.49	0.012	C_41_H_72_NO_11_P	[M + H]+	4.44
PS(34:4)	772.44	772.47	0.036	C_40_H_70_NO_11_P	[M + H]+	4.70
PS(35:0)	778.56	778.55	0.001	C_41_H_80_NO_10_P	[M + H]+	4.97
PS(35:5)	768.48	768.48	0.001	C_41_H_70_NO_10_P	[M + H]+	4.70
PS(36:7)	778.44	778.46	0.025	C_42_H_68_NO_10_P	[M + H]+	4.86
PS(38:3)	814.56	814.55	0.001	C_44_H_80_NO_10_P	[M + H]+	5.11
PS(36:6)	796.44	796.47	0.036	C_42_H_70_NO_11_P	[M + H]+	4.80
PS(39:8)	834.48	834.49	0.012	C_45_H_72_NO_11_P	[M + H]+	3.86
PS(34:0)	766.56	766.55	0.001	C_40_H_80_NO_10_P	[M + H]+	4.49
PS(38:9)	802.44	802.46	0.025	C_44_H_68_NO_10_P	[M + H]+	5.40
PS(37:7)	808.44	808.47	0.036	C_43_H_70_NO_11_P	[M + H]+	4.83
PS(37:6)	810.48	810.49	0.012	C_43_H_72_NO_11_P	[M + H]+	4.80
PE(34:6)	724.44	724.45	0.015	C_39_H_66_NO_9_P	[M + H]+	5.13
PE(36:8)	748.44	748.45	0.015	C_41_H_66_NO_9_P	[M + H]+	4.60
PE(32:3)	702.48	702.47	0.010	C_37_H_68_NO_9_P	[M + H]+	5.23
PE(36:5)	722.52	722.51	0.008	C_41_H_72_NO_7_P	[M + H]+	5.08
PE(32:5)	682.44	682.44	0.004	C_37_H_64_NO_8_P	[M + H]+	4.07
PE(40:5)	780.6	780.59	0.010	C_45_H_82_NO_7_P	[M + H]+	5.01
PE(40:8)	788.52	788.52	0.003	C_45_H_74_NO_8_P	[M + H]+	4.39

LC–MS Identified metabolites Rt-Retention time, Input mass, matched mass, Ion and their formula.

*Cer* ceramides, *PC* phosphatidylcholine, *PI* phosphatidyl inositol, *SM* sphingomyelin, *PS* phosphatidylserine, *PE* phosphatidyl ethanolamine.

### Analysis of differentially expressed metabolites

**Figure 2:**
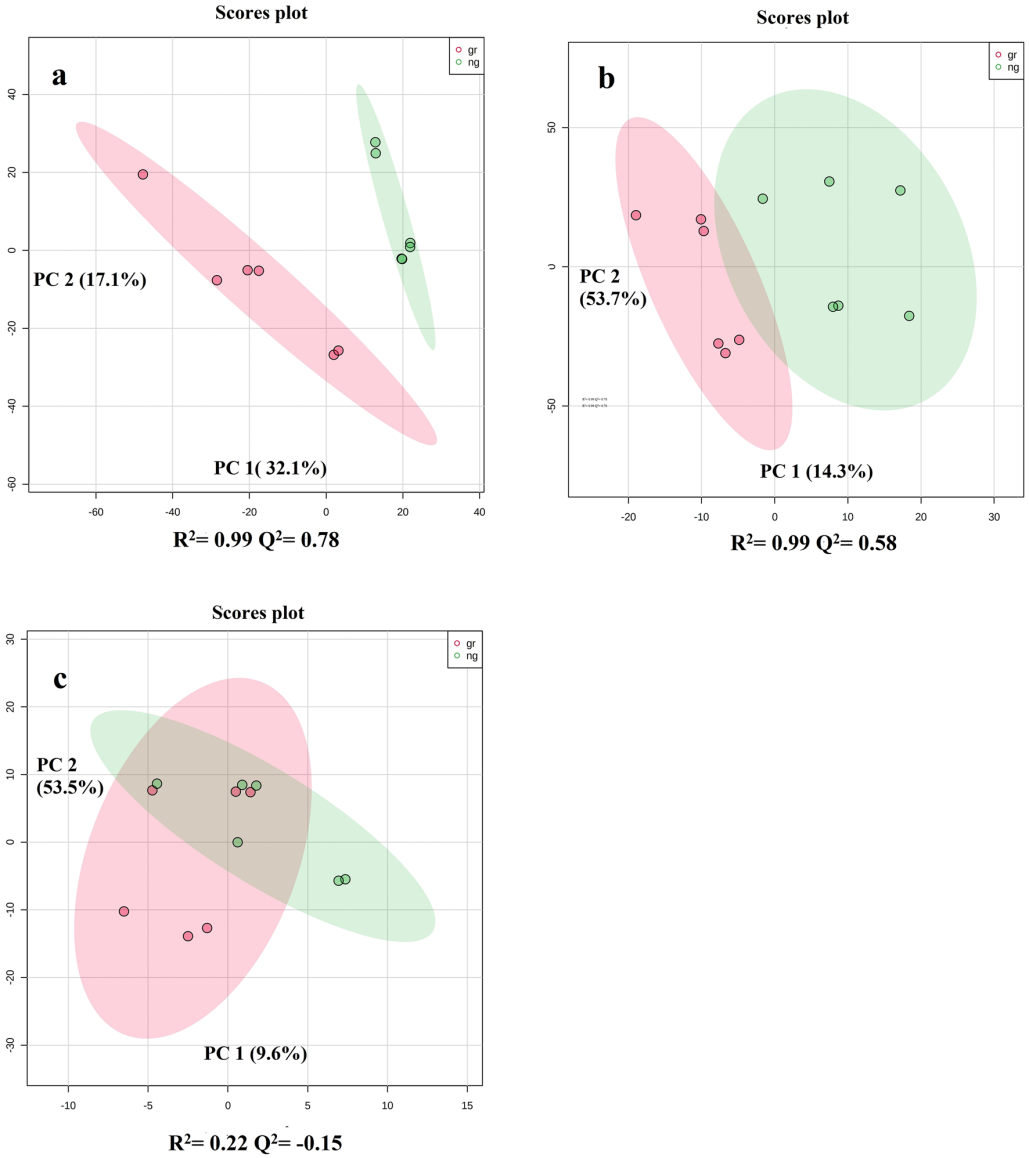
Partial least squares discriminate analysis (PLS-DA). (**a**) Score plots of acetonitrile precipitation of LC–MS data. (**b**) Score plots of methanol phase of LC–MS data. (**c**) Score plots chloroform phase of LC–MS data.

**Figure 3:**
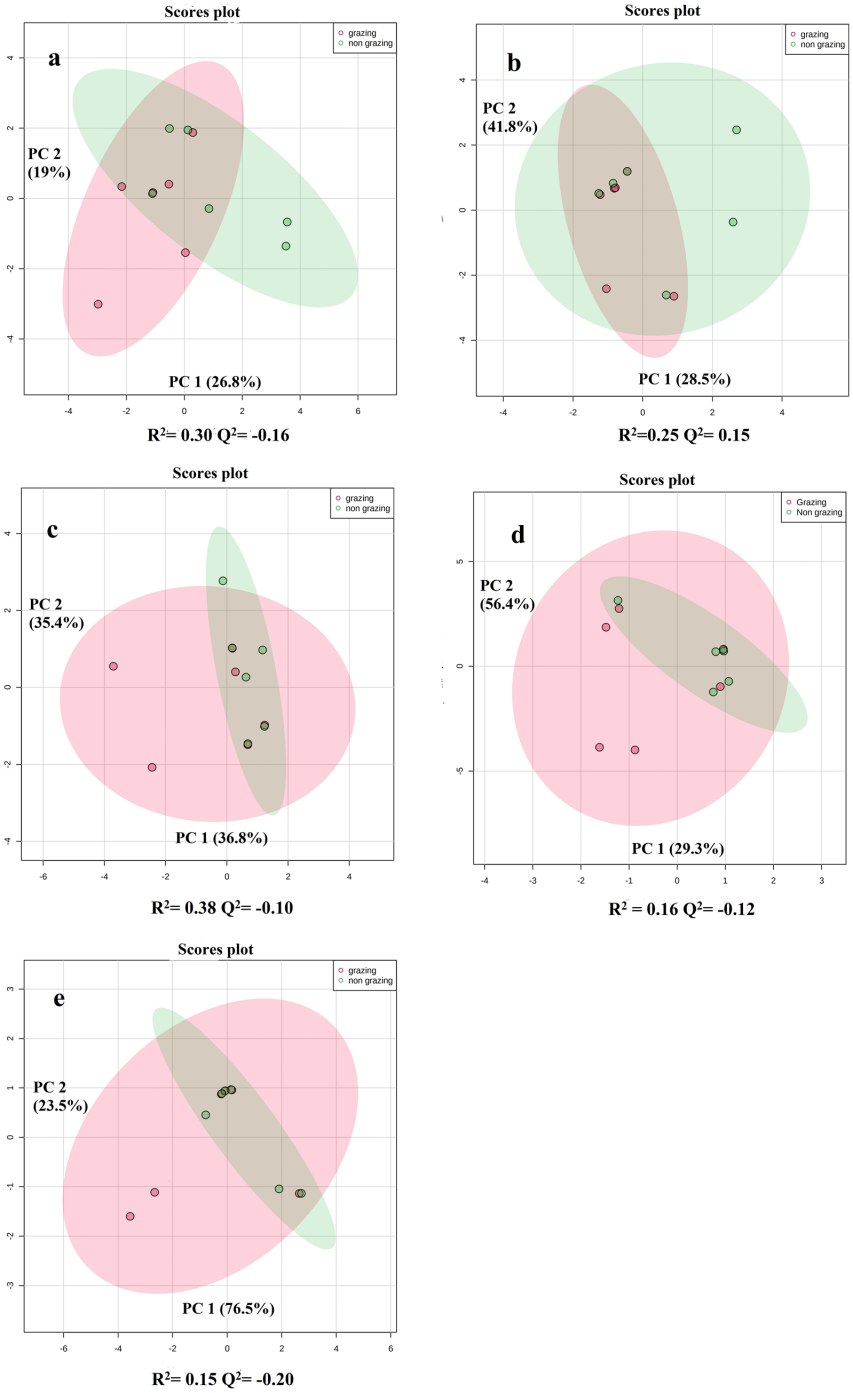
Score plot of PLS-DA model of (**a**) ceramides, (**b**) phosphatidylcholine (PC), (**c**) phosphatidylserine (PS), (**d**) phosphatidylethanolamine (PE) and (**e**) sphingomyelin (SM) of grazing and non-grazing cows. *PLS-DA* partial least square dimension analysis.

The partial least squares discriminate analysis (PLS-DA) of methanol phase, chloroform phase and acetonitrile precipitations were performed separately. PLS-DA of acetonitrile precipitation and methanol phase showed the difference between the milk of grazing and non-grazing cows (Fig. 2). Meanwhile, the PLS-DA plots of the chloroform phase showed partial overlapping between grazing and non-grazing cows. A ‘leave-one-out cross-validation’ method was performed to analyze the PLS-DA model. Q^2^ values of methanol phase and acetonitrile precipitation method were 0.58 and 0.78, respectively (Fig. 2). Q^2^ value of chloroform phase was 0.15. The Q^2^ values for individual lipid classes were also analyzed in the present study viz*.* ceramides, phosphatidylcholines, phosphatidylserines, phosphatidylethanolamines and sphingomyelins (Q^2^ values were 0.16, 0.15, 0.10, 0.12 and 0.20, respectively; Fig. 3). Q^2^ value more than 0.30 is fit for differential expression of metabolites study. Further, methanol:chloroform and acetonitrile precipitation methods were analyzed for differential metabolites expression. Our results showed that grazing has a major effect on milk polar metabolites as compared to milk lipids.

**Figure 4:**
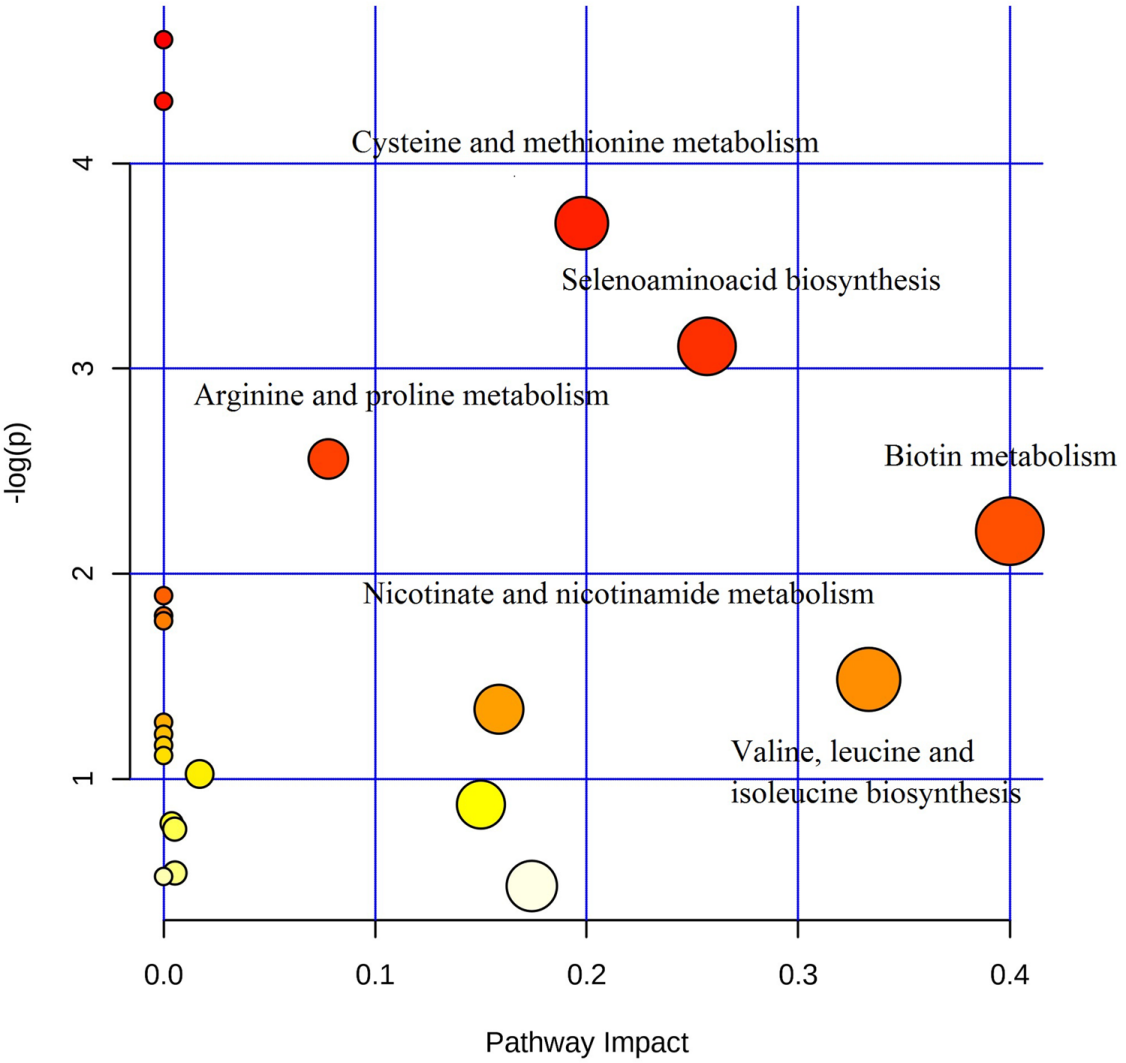
Pathway analysis of acetonitrile precipitation method. X axis indicates pathway impact and Y axis indicates pathway enrichment. Larger and darker rounds indicate the pathway enrichment and high pathway impacts.

Pathways analysis of acetonitrile precipitation (Fig. 4 and Table 2) resulted in the identification of differentially regulated pathways between the two groups. Biosynthesis and metabolism of cysteine, methionine, selenoamino acid, biotin, valine, leucine, isoleucine, nicotinate, nicotinamide, alanine, aspartate and glutamate were found to have high impact value i.e., above 1. Based on the impact value and significant p-value cysteine, methionine and selenoamino acid metabolism were found to be significantly differentially regulated. Further, we analyzed the LC–MS data for fold change in polar metabolites (methanol:chloroform) above 1.4-fold (Table 3) and acetonitrile precipitation fold change above 1.4 (Table 4).

**Table 2:** Milk metabolites pathway analysis.

Pathway name	Total comps	Hits	P	− log(p)	Impact
Thiamine metabolism	7	2	0.012	4.373	0.0
Aminoacyl-tRNA biosynthesis	64	5	0.021	3.817	0.0
Cysteine and methionine metabolism	28	3	0.033	3.397	0.197
Selenoamino acid metabolism	15	2	0.055	2.893	0.256
Arginine and proline metabolism	44	3	0.102	2.281	0.077
Biotin metabolism	5	1	0.123	2.095	0.4
Taurine and hypo taurine metabolism	7	1	0.168	1.783	0.0
One carbon pool by folate	8	1	0.189	1.662	0.0
Glycine, serine and threonine metabolism	32	2	0.199	1.611	0.0
Valine, leucine and isoleucine biosynthesis	11	1	0.251	1.380	0.333
Nicotinate and nicotinamide metabolism	13	1	0.289	1.238	0.158
Histidine metabolism	14	1	0.308	1.176	0.0
Pantothenate and CoA biosynthesis	15	1	0.326	1.119	0.0
Folate biosynthesis	16	1	0.344	1.066	0.0
beta-Alanine metabolism	17	1	0.361	1.017	0.0
Fructose and mannose metabolism	19	1	0.394	0.930	0.017
Alanine, aspartate and glutamate metabolism	23	1	0.455	0.786	0.15

Total Cmpd = total number of identified compoundsin the pathway; hits are the matched compounds of user uploaded data; *P*-value was calculated through the enrichment analysis; Impact value was calculated through pathway topology analysis.

**Table 3:** LC–MS (methanol:chloroform) method differentially regulating polar metabolites in grazing and non-grazing animals.

Metabolomic pathway	Compound name	Adduct	Fold change	log2(FC)
Amino acid	Phenylalanine	[M + H]+	0.003	− 8.099
Amino acid	Selenocystine	[M + H]+	3.286	1.716
Pyrimidine metabolism	Uridine diphosphate acetyl galactosamine 4 sulfate	[M + H]+	0.350	− 1.512
Carbohydrate	Ribose 1,5 bisphosphate	[M + H]+	0.415	− 1.268
Amino acid	l-lysine	[M + H]+	2.27	1.182
Purine metabolism	Cytidine.2, 3cyclicphosphate	[M + H]+	0.4932	− 1.019
Amino acid	Glycyl Methionine	[M + H]+	0.514	− 0.957
Amino acid	Zinc methionine sulfate	[M + H]+	0.518	− 0.945
Carbohydrate	Maltohexaose	[M + H]+	0.525	− 0.928
Amino acid	Valyl Valine	[M + H]+	0.554	− 0.850
Vitamin	Cyanocobalamin	[M + H]+	0.573	− 0.802
Carotenoids	Beta carotenal	[M + H]+	1.651	0.723
Purine metabolism	Inosine triphosphate	[M + H]+	0.618	− 0.693
Purine metabolism	Succinoadenosine	[M + H]+	1.522	0.606
Purine metabolism	Thioguanosine monophosphate	[M + H]+	1.416	0.501

**Table 4:** LC–MS identified (acetonitrile precipitation) differentially regulated polar metabolites in grazing and non-grazing animals.

Metabolomic pathway	Compound name	Adduct	Fold change	log2(FC)
Amino acid	Leucyl-Leucine	[M + H]+	0.135	− 2.885
Amino acid	Tyrosyl-Threonine	[M −H ]−	6.124	2.611
Amino acid	Histidinyl-Cysteine	[M + H]+	4.180	2.031
Amino acid	Threoninyl-Aspartate	[M + H]+	0.248	− 1.984
Carbohydrate	Fucose 1-phosphate	[M + H]+	0.261	− 1.899
Purine metabolism	1-Methyladenine	[M − H]−	3.476	1.738
Amino acid	Isoleucyl-Hydroxyproline	[M + H]+	0.293	− 1.717
Amino acid	l-Cysteine	[M − H]−	3.082	1.591
Amino acid	Aspartic acid	[M + H]+	2.998	1.581
Amino acid	Glutaminyl valine	[M + H]+	2.912	1.549
Amino acid	Arginyl arginine	[M + H]+	0.378	1.535
Carbohydrate	l-arabinose	[M + H]+	0.405	− 1.250
Carbohydrate	Ribose 1,5-bisphosphate	[M + H]+	0.454	− 1.130
Amino acid	Serylmethionine	[M + H]+	2.032	1.102
Amino acid	Glycyl-glycine	[M − H]−	2.021	1.015
Amino acid	5-Hydroxylysine	[M + H]+	1.976	0.978
Amino acid	Aspartyl-Tyrosine	[M + H]+	0.516	− 0.834
Amino acid	Threoninyl-Proline	[M − H]−	1.706	0.760
Amino acid	Selenocysteine	[M − H]−	1.667	0.752
Amino acid	Glycyl-Arginine	[M + H]+	0.650	− 0.704
Amino acid	Leucyl-Histidine	[M − H]−	0.689	− 0.572
Amino acid	l-Proline	[M + H]+	1.432	0.516

The fold change of metabolites was calculated by dividing grazing concentration and non-grazing concentration. Selenocysteine showed the highest fold change (3.29) in grazing animals. Amino acid lysine was higher in grazing animals. Beta carotenol (Vit A precursor) was found to be up-regulated in grazing animals at a fold change of 1.65. The concentrations of nucleosides such as thioguanosine monophosphate and succino adenosine were increased in the grazing animals around 1.5-fold change compared to the non-grazing animals. Meanwhile, uridine diphosphate, cytidine 2, 3 cyclic phosphates and inosine triphosphate were found to be down regulated in grazing animals.

More number of amino acids were found to be differentially regulated in acetonitrile precipitation as compared to the methanol:chloroform method. Tyrosyl-threonine showed the highest fold change of 6.12 followed by histidinyl-cysteine with a fold change of 4.18. l-cysteine, glutaminyl valine and aspartic acid showed higher concentration in grazing animals. Ribose 1,5-bisphosphates, the common metabolite, concentration was found to be 0.4 in both the methods. Leucyl-leucine and methionyl-serine showed the least concentrations in grazing animals as compared to non-grazing animals. Carbohydrates such as fucose 1-phosphate and l-arabinose were found to have a lower concentration in grazing animals.

## Discussion

In the present study, differentially expressed milk metabolites were identified in cows reared under pasture-based and stall-fed feeding systems based on two different methods (acetonitrile precipitation and methanol: chloroform) along with their associated pathways. The data acquisition standards and recommendations for untargeted metabolomics have evolved, from times of simple way of blank profile comparison based quality control (QC) strategies^20–22^ to including multiple injections of the pooled-QC samples along with the sample acquisition batches^19^. We acquired data before the arrival of pooled-QC strategies and therefore, used blank runs as a means to prevent sample-carryover and to differentiate the sample run profiles from the blanks. However, the metabolite assignment was carried out at both the precursor and fragment levels, using the MS2Compound tool, yielding a set of high confidence assignment of the metabolites. Most of the amino acids in Malnad Gidda milk were detected from the acetonitrile precipitation method. Methanol:chloroform method was found to be more effective for lipidomic study as it resulted in maximum lipid metabolites coverage compared to acetonitrile precipitation.

Our study showed that grazing animal milk was rich in beta carotene. Fresh pastures naturally have a high concentration of beta carotene^23,24^ whereas, the ensiling and other processing of feeds for livestock, generally deplete many carotenoids due to which non-grazing cow’s milk is naturally low in beta carotene^25^. Therefore, beta carotene could be used as an important biomarker for the identification of milk from fresh pasture-fed dairy cows^26^. Similarly, biotin and niacinamide were found to be up-regulated in grazing cow’s milk (fold change 1.3). Biotin is a cofactor for propionyl-coenzyme A carboxylase and pyruvate carboxylase. Biotin and niacin play a major role in oxidation–reduction reactions^27^ and gluconeogenesis^28^. Niacin (nicotinic acid), nicotinamide (niacinamide) and nicotinamide riboside are the three forms of vitamin B_3_. All the three forms get converted into nicotinamide adenine dinucleotide (NAD) and nicotinamide adenine dinucleotide phosphate (NADP)^29–31^. Further, cobalamin was found to be down-regulated in the milk from the grazing animals. Foods of ruminant origin are a very good source of cobalamin (vitamin B_12_). Rumen bacteria produce cobalamin which is secreted via milk or stored in the liver or muscles^32^.

The majority of the amino acids showed differential regulation between grazing and non-grazing dairy cows. The molecular and physiological mechanisms of an animal were found to be mainly affected by the feeding systems viz*.,* grazing and non-grazing. This might lead to the alteration in the metabolic pathways and their associated metabolites. The two mentioned feeding systems significantly affected the metabolism of cysteine, methionine and selenoamino acid s (P < 0.05). Sun et al*.*^12^ have reported that two different diets (corn stover and alfalfa hay) had a significant effect on glycine, serine and threonine metabolisms in milk. In grazing animals cysteine, methionine and seleno amino acid metabolic pathways were found to be upregulated. Cysteine contains a thiol group, plays a major role in energy metabolism and antioxidant activity, and has an affinity towards redox reactions^33^. Selenocysteine showed the highest fold change (3.286) in the milk of grazing animals. This might be due to the upregulation of selenoamino acid metabolism (P < 0.05) in grazing animals. Occasional toxicity had been frequently observed in cows grazing in selenium-rich pastureland. Glycine was found to be up-regulated in the milk of grazing cows. The finding is in agreement with Magan et al.^34^ who reported that pasture-derived cow’s whey showed a higher concentration of glycine compared to TMR cows. Glycine is a non-essential amino acid primarily involved in collagen synthesis. It has limited work in other metabolic pathways and protein production^35^. Phenylalanine and glycyl methionine were down-regulated in grazing animals. Phenylalanine is an essential amino acid, which is important for the production of tyrosine, epinephrine and dopamine. In stress conditions, phenylalanine and tyrosine get involved in the synthesis of tyramine, dopamine and adrenaline^36^. Arginine is a precursor for glutamate, polyamines, proline and creatine and plays a major role in metabolic processes^37^**.** Leucine and valine were down-regulated and proline was up-regulated in the milk from grazing animals. O’Callaghan et al.^6^ have also reported that down-regulation of amino acids such as leucine and valine in the milk of grazing animals, but contradictorily, they also reported proline to be down-regulated in grazing animals. Amino acid L-glutamate is responsible for proline biosynthesis. Valine plays a major role in the synthesis of a globular protein, where it forms a nonpolar center covered by a polar part^38^. It is also involved in insulin secretion and other metabolic functions^39^.

Carbohydrates such as maltose, ribose 1,5-biphosphate, arabinose and fucose 1-phosphate were found to be down-regulated in the milk of grazing cows. O’Callaghan et al*.*^6^ have also reported that disaccharide D-maltose was down-regulated in pasture-based milk. This might be due to the feeding of concentrates to non-grazing animals. Ribose 1-phosphate is the precursor for ribose 1,5-bisphosphate which is catalyzed by phosphoglucomutase^40^. Fructose 6 phosphates/fructose 1, 6 bisphosphate cycles get regulated by ribose 1,5-bisphosphate in liver^41^. Fucose is a complex heterooligosaccharides. The GDP-Fucose synthesis pathway is enabled by GDP-fucose pyrophosphorylase where fucose kinase and fucose-1-phosphate are the intermediate metabolites. Fucose-containing glycans play a major role in host-microbe interactions and numerous ontogenic events^42^.

The Non-Protein Nitrogen (NPN) part of milk consists of nucleosides, nucleotides and nucleobases. Nucleotides are synthesized via the salvage pathway and hence are not essential nutrients^43^. Uridine diphosphate acetyl galactosamine 4 sulfates were found to be down-regulated in grazing animals. O’Callaghan et al.^6^ have also reported the down-regulation of uridine in grazing animals. Fresh forage and hay-based feeding systems result in a marked elevation of uridine in cows. This might be due to an alteration in the nucleotide metabolism in animals^13^. In cow milk, nucleotides and nucleosides concentration, especially uridine and uridine 5-monophosphate (UMP) was found to be high in early lactation^44^. As the lactation advanced, the nucleotide concentration decreased first and then remained stable after 21 days of lactation. On the contrary, cytidine 5 -monophosphate (CMP) and cytidine always remained high^44^.

The lipidomic profile of milk is an essential factor for dairy farming and the most technological quality of raw milk^13^. In the present study, lipids showed less differential regulation between grazing and non-grazing cows as compare to polar metabolites. Lipids had a high nutritional impact in milk but their concentrations were not altered as compared to polar metabolites. Our results indicated that polar metabolites played a major role in feed-based metabolomics study.

The untargeted metabolomics analysis sheds light on the various metabolites that are differentially regulated as a result of feeding patterns in cows. However, the current study suffers from the limitation of not using the quality control samples to monitor the data acquisition and standards to confirm metabolite signatures, as this data was acquired before such strategies have become a standard practice only in recent times. However, blank runs were compared with the sample runs to eliminate carryover and differentiate the sample-profile from the blank runs. Also, keeping in mind the quality of the metabolite assignment in the absence of metabolite standards, we used a fragment-level identification using MS2 Compound, which showed coherence in the metabolite identification at both the MS1 and MS/MS levels. The assignment of metabolites at the fragment-level adds high confidence to the reporting of the differentially altered metabolites that are identified in the study. This metabolomics study could provide new insight into understanding the role of polar metabolites with comparison to lipids in different feeding systems.

## Conclusions

Our study indicated that the pasture (grazing) based feeding system had brought significant changes in the polar metabolite profile in the milk rather than the non-polar lipidomic profile in Malnad Gidda cows. The metabolite identification at the MS/MS level serves as a major factor in determining the quality of the metabolite assignment and the subsequent data analysis. Unlike nucleosides, carbohydrates and vitamin derivatives; majority of the amino acids were found to be differentially regulated between grazing and non-grazing cows. This study reports the alterations in the metabolites like tyrosyl-threonine, histidinyl-cysteine, 1-methyladenine, cysteine and selenocysteine which showed a huge variation in the abundance and found to be in higher amount in the milk of grazing cows versus that of non-grazing. Further, the variation in lipidomic profile was comparable between the two groups. However, further studies to analyze the effect of the feed with different pasture composition and concentrate supplementation could offer significant insight on milk metabolites to strengthen the methodology.

## Methods

### Collection of samples

Milk samples were collected from a total of twelve healthy, non-pregnant multiparous Malnad Gidda cows in early lactation reared under grazing and non-grazing conditions in the Western Ghats region of Karnataka. All experimental protocols were accepted by the Institutional Animal Ethics Committee, ICAR- National Dairy Research Institute, Southern Regional Station, Bengaluru (F.No. CPCSEA/IAEC/SRS-ICAR-NDRI-2019/No.18, dated 03.01.2019). Six cows were selected for the collection of milk under grazing conditions. Pasture based green grasses and forages were the main feeds of the grazing cows. The details of forage resources available in Malnad Gidda native tract were *Chloris gayana* (DM-20.00), *Cynodon dactylon* (DM-32.08) and *Eleucine indica* (DM-34.38)^18^. The days in milk collection, parity and milk yield details are given in supplementary Table S3. Six cows were selected for the collection of milk under non-grazing condition which were fed with 1.5 kg paddy straw, maize fodder 4 kg and 400 g groundnut powder. The dry matter content was 87% and 23.2% for paddy straw and maize fodder, respectively. The grazing and non-grazing animals were milked at 6.00 PM. A routine hand milking procedure was followed for the collection of milk samples. California Mastitis Test (CMT) was performed for screening of clinical and subclinical mastitis. The milk samples were stored at -80 °C immediately after collection.

### Isolation of metabolites

**Figure 5:**
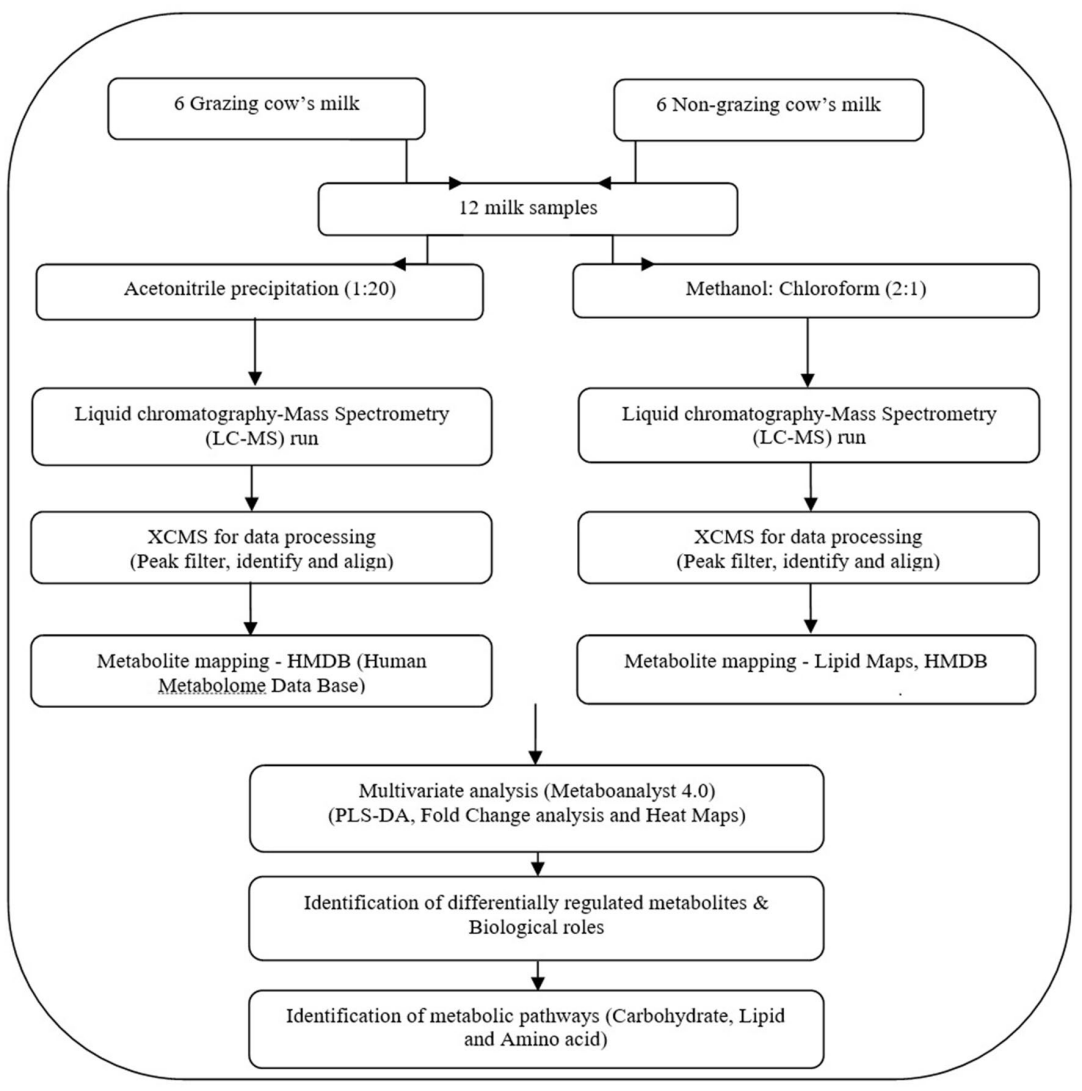
Overview of milk metabolomics investigation on grazing and non-grazing Malnad Gidda cows. *HMDB* Human Metabolome Data Base, *PLSDA* partial least square—dimension analysis.

All methods and experiments were carried out in accordance with relevant guidelines and regulations. In order to get maximum metabolomics coverage, we performed the experiments based on two different methods (acetonitrile precipitation and methanol:chloroform) for isolating the milk metabolites. Folch method (methanol:chloroform) was used to study the lipid profile, which had been a golden standard method to study the lipid profile in biological samples^45^. Overview of the plan of work of milk metabolomics on grazing and non-grazing Malnad Gidda cows is shown in Fig. 5.

Milk samples were analyzed for their protein amounts using bicinchoninic acid assay (BCA assay), which was confirmed by resolving the samples on a 10% SDS-PAGE gel. The protein amount data were used for the normalization of the sample volumes before the metabolite extraction, using Folch’s method. Briefly, sample volume equivalent to about 1 mg of protein amount was used for the metabolite extraction and the sample volumes were made equal before extraction to a final volume of 47 µL. The extraction solvent consisting of chloroform:methanol and water was used. To the samples, chloroform:methanol (ratio of 2:1) mixture of 950 µL was added, followed by vortexing for 10 min at room temperature and sonicated using a water-bath sonicator for 10 min. To this, water at 0.2 times the volume of chloroform:methanol mixture, i.e., 190 µL of LC–MS grade water was added. The extraction mixture was further vortexed for 5 min and left to stand-alone undisturbed at room temperature for 5 min, to induce phase-separation. The mixture was then centrifuged at 10,000*g* for 15 min at 4 °C. The separated phases, upper and lower, were carefully transferred to separate tubes and dried using SpeedVac, overnight. The interphase containing the precipitated proteins was discarded. The samples were stored at − 20 °C until mass spectrometry analysis. Before mass spectrometry analysis, the upper phase was reconstituted in 200 µL of 0.1% formic acid in the water, while the lower phase was reconstituted 200 µL in 0.1% formic acid in 90% acetonitrile. The samples were further diluted to 1:5 in the respective solvents, and 10 µL of the sample was injected.

We also extracted the metabolites by precipitating protein from milk (volume of milk was 1 mg equivalent weight to protein) by adding a 1:20 ratio of milk and Acetonitrile. Followed by 2 min vertex at RT and centrifugation at 10,000*g* at 4 °C for 10 min. The supernatant was taken into the fresh tube and dried in a speed vac. The dried samples were reconstituted in 100 µl of 0.1% Formic acid in MS grade water.

### LC–MS/MS analysis

QTRAP 6500 mass spectrometer, ABSciex was coupled with Agilent 1290 Infinity II liquid chromatography system, with C18 RRHD Zorbax column (20 × 150 mm, 1.8 μm particle size), Agilent, ZIC HILIC (150 mm × 4.6 mm, 5 μm particle size), Merck, and C18 RRHD Zorbax column (21 × 50 mm, 1.8 μm particle size), Agilent as the analytical columns. Analyst software version 1.6.3 with the Analyst Device Driver was used to set the parameters for the analysis. The separation of the metabolites was carried out using 60 min and 40 min separately for the LC method. The reverse-phase liquid chromatography (RPLC) method was used with solvents, A: 0.1% formic acid in Milli-Q water, and B: 0.1% formic acid in 90% acetonitrile. The 60 min gradient used for the RPLC method was as follows; t = 0–5 min, B = 2%; t = 20 min, B = 30%; t = 35 min, B = 60%; t = 46–52 min, B = 95%; t = 54–60 min, B = 2%. The HILIC method was used with solvents, A: 0.1% formic acid in 90% acetonitrile and B: 0.1% formic acid in Milli-Q water. The 60 min gradient used for the HILIC method was as follows; t = 0–5 min, A = 2%; t = 20 min, A = 30%; t = 35 min, A = 60%; t = 46–52 min, A = 95%; t = 54–60 min, A = 2%.

The solvent A was 0.1% formic acid in Milli Q water and solvent B was 0.1% formic acid in 90% acetonitrile, the flow rate was set to 0.3 mL/min and the gradient was 2% B for 2 min, 2–30% B for 8 min, 30–98% B for 20 min, 98% B for 5 min, 98–2% B for 3 min, and 2% B for 2 min.

The mass spectrometry data acquisition was carried out with Information Dependent Acquisition (IDA) method in low mass mode. The IDA method was built using the Enhanced Mass Spectra (EMS) to Enhanced Product Ion (EPI) modes. The top five spectra from the EMS mode were used for analysis in the EPI (MS/MS) mode; using high energy Collisionally Induced Dissociation (CID). The metabolite data were acquired in positive (data/ions/polarities) at 4500 V and − 4500 V, respectively, with a probe temperature of 450 °C. The compound parameters were set at a Declustering Potential (DP) of 75 V, Collision Energy (CE) of 45 V. The column conditioning, before and after every sample was included within the LC gradient used for the data acquisition, resulting in a total of 10 min of re-equilibration for ensuring the maintenance and reproducibility^21,22^. The data was acquired in technical triplicates, as different batches consisting of intermediate blank runs, which were injected after every triplicate run of the samples to prevent carryover.

### Data analysis using XCMS

LC–MS/MS raw data format ‘.wiff’ files from Analyst software was converted to ‘.mzML’ using MS Convert^46^. Data processing was carried out using the XCMS online tool^47^, using the Multigroup analysis option. Feature detection was carried out with a centwave algorithm, with 10 ppm mass error for consecutive scans, peak width of 10–30 s and a signal/noise ratio of 5. The alignment was carried out with a retention time deviation of 20 s and a minimum fraction of samples as 0.6 and a mzdiff of 0.15. Data was searched for isotopes and adduct mass error of 5 ppm. The m/z features from XCMS were searched against Lipid maps (https://lipidmaps.org/) and Human metabolome database (www.hmdb.ca/).

The blank run raw files from each data acquisition batches were used to assess the quality of the sample data acquisition. XCMS analysis of the blank runs along with the actual samples was carried out and the aligned peak intensity table was used in the Metaboanalyst platform to assess the clustering of the samples and the blank runs from different batches, as demonstrated by Martinez-Sena et al.^19^.

### Metabolite assignment at MS/MS-level

Alternatively, the .mzML raw data files were used for processing in MZMine 2.5^48^, for peak alignment and extracting the fragment-level information. The raw files were processed for mass detection at MS1 and MS/MS levels, using the centroid mode, with minimal peak intensities of 1.0E3 and 1.0E1, respectively. The m/z feature-list building was performed with MS/MS peak list builder algorithm, which selects only the precursors that have MS/MS-level information. Peak extension algorithm was used on the feature lists, which was further deconvoluted using chromatogram deconvolution using the noise-amplitude algorithm and a minimum peak intensity of 1.0E3, a noise peak intensity of 1.5E2, and m/z and rendition time tolerance of 0.1 Da and 1 min, for MS/MS pairing. The deconvoluted feature lists were then processed for isotopic peak grouping with a maximum charge state of 4, with a respective isotopic m/z tolerance of 0.25 Da, and a retention time tolerance of 0.2 min. The deisotoped feature lists from the replicate runs of the grazing and the non-grazing samples were aligned using the Join-Aligner algorithm, with an m/z tolerance of 0.05 Da, retention time tolerance of 0.4 min, and m/z and retention time thresholds of 70% and 30% respectively. Further, a gap-filing algorithm was used on the aligned feature list with an m/z tolerance of 0.05 Da, and retention time tolerance of 0.5 min and duplicate peaks were removed. The results were exported as a quantification table with the peak area, retention time, only for the respective m/z features with MS/MS information. Similarly, the fragment m/z information and the respective fragment intensities of the precursors were exported as .mgf files.

The .mgf files containing the precursor and fragment information was used for metabolite assignment, based on the fragment-level matches, using the in-house MS2Compound tool (https://sourceforge.net/projects/ms2compound/). The metabolites from the Human Metabolite Database (www.hmdb.ca), were downloaded and theoretically fragmented using the Competitive Fragmentation Modeling-ID (CFM-ID) tool^49^, and were used as the background database for searching the milk metabolites. The MS2Compound matches the respective metabolites to the precursor m/z, based on the charge and adducts, which are then matched to the experimental fragments and the CFM-ID-generated theoretical fragments. The metabolite assignment is given an *mS*-score and a Rank, based on the precursor and fragment m/z match, fragment intensity match, and several fragments matched to the precursor. A precursor tolerance of 0.05 Da, a fragment tolerance of 0.5 Da, and a cut-off of a minimum of two fragment matches were used for metabolite search against the database. The metabolite assignment to a respective m/z was considered based on, a minimum of two fragment-level matches, and identifications with the highest *mS*-score and top Rank.

### Statistical analysis

Metaboanalyst (https://www.metaboanalyst.ca/) was used for data normalization and statistical analysis. Normalization was done by normalization by a median, log transformation and autoscaling or paretoscaling. Metaboanalyst 4.0 software was used for Partial least squares discriminant analysis (PLS-DA). The ‘leave-one-out cross-validation’ method was performed to analyse the PLS-DA model. The metabolites and their associated pathway were identified through Metaboanalyst 4.0 online software against the *Bos taurus* pathway library.

## Supplementary Information

Supplementary Figure.

Supplementary Table 2.

Supplementary Table 3.
