# Supplementary figures and images for: Differences in milk metabolites in Malnad Gidda (Bos indicus) cows reared under pasture-based feeding system
Source: Sci Rep. 2021 Feb 2;11:2831. doi: 10.1038/s41598-021-82412-z (PMC7854684; doi:10.1038/s41598-021-82412-z)

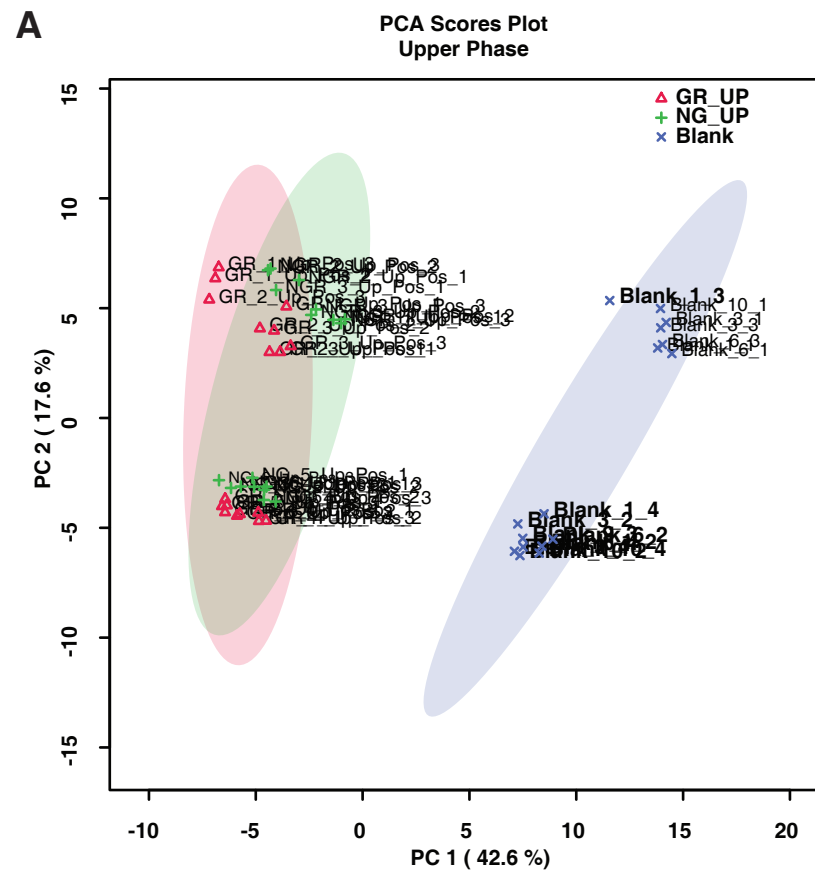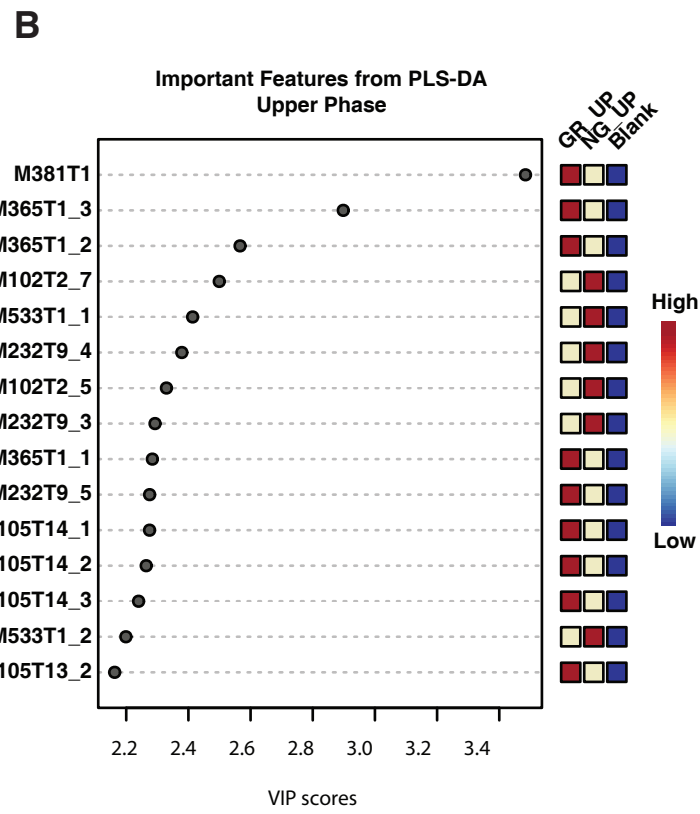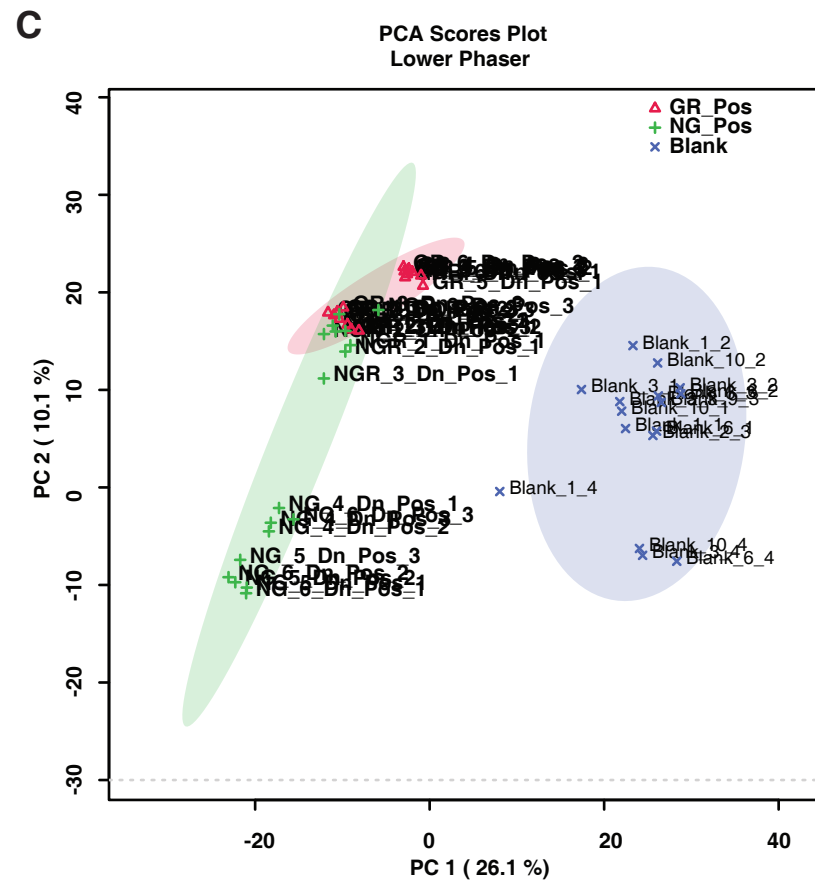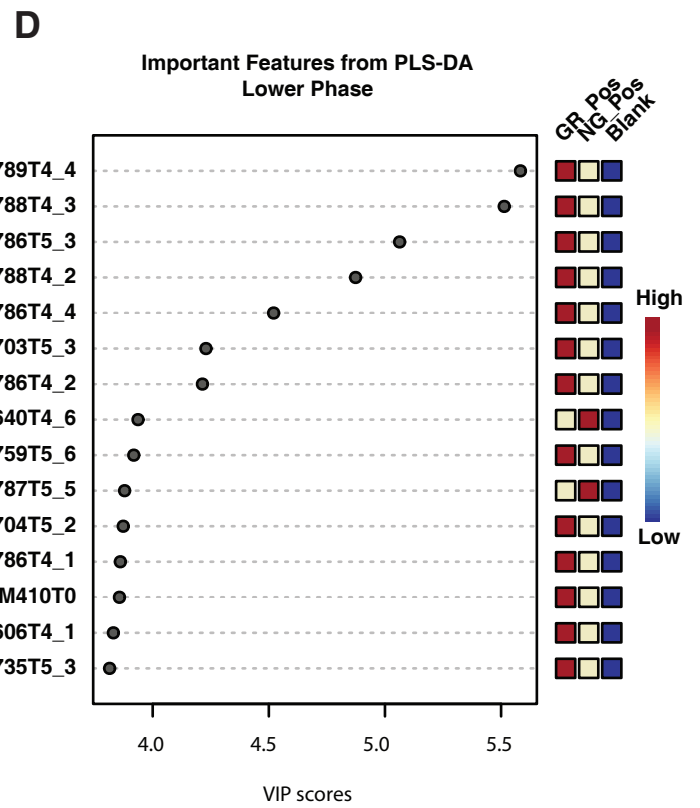

Supplement: Supplementary file 1 — Supplementary Figure. [file 41598_2021_82412_MOESM1_ESM.pdf]
