# Supplementary material for: Differences in milk metabolites in Malnad Gidda (Bos indicus) cows reared under pasture-based feeding system
Source: Sci Rep. 2021 Feb 2;11:2831. doi: 10.1038/s41598-021-82412-z (PMC7854684; doi:10.1038/s41598-021-82412-z)
Supplement: Supplementary file 2 — Supplementary Table 2. [file 41598_2021_82412_MOESM2_ESM.pdf]

Differences in milk metabolites in Malnad Gidda (*Bos indicus*) cows reared under pasture-based feeding system

M. Ashokan[1], Kerekoppa P. Ramesha[1*], Sweta Hallur[2], Gayathree Karthikkeyan[2], Ekta Rana[1], N. Azharuddin[1], S. Reshma Raj[1],S. Jeyakumar[1], A. Kumaresan[1], Mukund A. Kataktalware[1], D.N. Das[1] and T. S. KeshavaPrasad[2,*]

[1]ICAR- National Dairy Research Institute, Adugodi, Bengaluru-560030, India. [2]CSBMM, Yenepoya Research Centre, Yenepoya (Deemed to be University), Mangalore-575007, India.

***Corresponding author**:

Kerekoppa P. Ramesha*, M.V.Sc, Ph. D
Principal Scientist and Head
Southern Research Station, ICAR-National Dairy Research Institute
Adugodi, Bangalore 560030, India
E-mail: kpragb@gmail.com; Contact Number: 080-25711209

T. S. Keshava Prasad, Ph.D.
Professor and Deputy Director
Center for Systems Biology and Molecular Medicine
Yenepoya Research Centre | Yenepoya (Deemed to be University)
University Road | Deralakatte | Mangalore 575018 | India
Email: keshav@yenepoya.edu.in | Mobile: +91-9972250102

Figure legend:

Supplementary Figure S1: Assessing the quality of data acquisition. Blank runs were analyzed for their clustering pattern upon comparison with the actual samples. (A) PCA scores plot for the sample and blank runs from the upper phase, (B) Important features identified form the upper phase based on PLS-DA method, (C) PCA scores plot for the sample and blank runs from the lower phase, (D) Important features identified form the lower phase based on PLS-DA method.

Supplementary Table S1: Metabolites identified in methanol: chloroform method

Metabolites	m/z ratio
Valyl-Valine	109.08
Phenylalanylphenylalanine	157.08
Adenosine thiamine triphosphate	377.04
Glycyl-Methionine	104.04
L-beta-aspartyl-L-aspartic acid	125.04
Uridine diphosphategalactose	284.04
Thiamine monophosphate	173.04
Selenocystine	336.96
Ribose 1,5-bisphosphate	156
Selenomethionine	198
Inosine triphosphate	255
Cobalt sulfate	78.48
Cyanocobalamin	678.84
Maltohexaose	496.2
3'-O-Methyladenosine	282.12
Diguanosine diphosphate	709.08
Iodine	127.92
Zinc methionine sulfate	309.9
Histidinyl-Cysteine	259.08
Thioguanosine 5'-diphosphate	230.52
Succinoadenosine	384.12

6-Thioguanosine monophosphate	380.04
Ferrocytochrome	301.08
Uridine diphosphate acetylgalactosamine 4-sulfate	344.52
Adenylylselenate	238.44
Thiamine pyrophosphate	212.52
6-Galloylglucose	391.08
Triphosphate	258.96
N-Methyl-L-proline	130.08
ADP-Ribosyl-L-arginine	716.16
Cob(II)alamin	664.8
NADP	744.12
Nicotinic acid adenine dinucleotide	332.52
Malyl-CoA	442.56
NADH	666.12
S-Cysteinosuccinic acid	119.52
Methionyl-Methionine	281.1
beta-Carotinal	209.16
Methyl glucosinolate	167.52
Deoxyuridine triphosphate	468.96
Cytidine 2',3'-cyclic phosphate	153.48
Fucose 1-phosphate	245.04
6-Thioguanosine-5'- O- triphosphate	522

Histidinyl-Glutamate	285.12
Coenzyme A	768.12
Zinc sulfate	160.92
ADP-ribose 1"-2" cyclic phosphate	311.52
Diadenosine pentaphosphate	917.04
N-Oleoyl GABA	184.68
Dimethyl diselenide	190.92
dCTP	234.48
3,5-Diiodo-L-tyrosine	217.44
Asparaginyl-Histidine	270.12
N-Formyl-L-aspartate	81.48
Asparaginyl-Glutamine	261.12
dTDP-4-acetamido-4,6-dideoxy-D-galactose	295.56
dADP	206.52
Adenosine 5'-pentaphosphate	334.44
Adenosylcobalamin	790.32
Acetyl-CoA	405.6
Oleoyl glycine	170.64
Fumarycarnitine	130.56
Deoxyadenosine triphosphate	492
4-Hydroxyphenylacetyl-CoA	451.56
histamine phosphate	308.04
Adenosyl cobinamide phosphate	659.76

N-Phenylacetylaspartic acid	126.54
L-lysine	122.04
Hexaglutamyl folate	544.2
Guanosine diphosphate adenosine	693.12
5-Carboxy-2'-deoxyuridine	137.04
dTDP-D-glucose	565.08

Supplementary Table S2: Metabolites identified in acetonitrile method

Metabolites	m/z ratio
L-Aspartic acid	377.225
L-Proline	425.25
Selenocystine	336.96
Methionyl-Serine	237.1
Methionyl-Methionine	281.1
L-Isoleucine	53.5
D-Leucine	132.1
Selenomethionine	198
Ribose 1,5-bisphosphate	156
Threoninyl-Aspartate	118.05
Isoleucyl-Methionine	132.075
Threoninyl-Isoleucine	233.15
Isoleucyl-Hydroxyproline	245.15

Molybdate	81.95
Histidinyl-Threonine	257.125
Tryptophyl-Lysine	167.1
5-Hydroxy-L-tryptophan	111.05
Ureidosuccinic acid	177.05
Pyrophosphate	178.95
Arsenocholine	165.025
Diguanosine tetraphosphate	435.025
Tyrosyl-Lysine	310.175
D-Arabitol	153.075
Cytosine	112.05
N-Ornithyl-L-taurine	240.1
Thiocysteine	154
Folic acid	442.15
Tyrosyl-Histidine	160.075
Histidinyl-Glycine	213.1
Thiamine monophosphate	345.075
Serylmethionine	119.05
Biotin	123.05
L-Alanyl-L-methionine	111.05
S-Adenosylmethionine	399.15
Lysyl-Methionine	278.15
Aspartyl-Aspartate	249.075

S-adenosyl-L-methioninate	399.15
N-Acetyl-L-phenylalanine	208.1
L-Cysteine	122.025
Aspartyl-Cysteine	237.05
Glutaminylvaline	246.15
Cysteinyl-Proline	219.075
Valyl-Valine	215.15
Uridine 2',3'-cyclic phosphate	305.025
Histidinyl-Cysteine	130.05
L-Thyronine	274.1
Leucyl-Leucine	123.1
Tryptophyl-Phenylalanine	352.175
Aspartyl-Tyrosine	297.1
Fucose 1-phosphate	245.05
Prolyl-Tyrosine	279.125
Glycyl-Arginine	232.15
Niacinamide	123.05
5-Hydroxylysine	161.1
UDP-L-arabinose	537.025
Cysteinyl-Cysteine	225.025
Arginylarginine	329.2
1-Methyladenine	150.075
Adenine	134.05

Selenocysteine	167.95
Leucyl-Histidine	267.15
Threoninyl-Proline	215.1
Tyrosyl-Threonine	267.1
Cysteinyl-Leucine	233.1
Lysyl-Proline	242.15
Leucyl-Hydroxyproline	243.125
Threoninyl-Cysteine	221.05
L-Threonine	118.05
N-lactoyl-Methionine	220.075
Glycyl-glycine	131.05

Supplementary Table S3: The days in milk collection, parity and milk yield details of animals

**Animal number**	**Days in milk**	**Parity**	**Milk yield in Kgs per day**
**Grazing animals**			
1	20	4	1.2
2	25	3	1.0
3	55	2	1.3
4	68	3	0.8
5	30	2	1.2
6	35	5	1.0
**Non-grazing animals**			
1	35	3	0.6
2	50	2	0.8
3	60	4	0.9
4	28	2	1.0
5	45	5	0.7
6	15	3	1.2
